# Flu RNA Vaccine: A Game Changer?

**DOI:** 10.3390/vaccines8040760

**Published:** 2020-12-14

**Authors:** François Meurens

**Affiliations:** INRAE, Oniris, BIOEPAR, 44300 Nantes, France; francois.meurens@oniris-nantes.fr; Tel.: +33-2-40-68-77-02

Influenza virus infection is a major One Health concern worldwide. Indeed, *Orthomyxoviridae* and more specifically *Alphainfluenzavirus* and *Betainfluenzavirus* are responsible for flu disease, which is mostly associated with respiratory and systemic clinical signs in various species including humans, pigs, horses, ferrets and birds [1]. Zoonoses involving influenza virus strains are common, and even clear evidence of bidirectional human–swine transmission has been reported [2]. A large-scale use of therapeutic approaches—such as oseltamivir phosphate, zanamivir, and newer drugs such as baloxavir marboxil—is not possible and could favor the emergence of antiviral drug resistances, the first line of defense against influenza viruses remains to be the vaccination of the exposed populations. However, vaccination against influenza viruses still presents several drawbacks, the main ones being a relatively low effectiveness and strain mismatches. Currently available vaccines in humans are 40–60% effective and offer poor protection or no protection against other strains, especially if they are from a different subtype of influenza virus [3,4,5,6,7,8]. Thus, there is undoubtedly room for improvement in the challenging world of flu vaccines.

A growing interest has been shown for a new generation of vaccination approaches using nucleic acids such as DNA or RNA. However, because of their suboptimal potency in early clinical studies [9] and the low but persistent risk of the integration of DNA sequences into the host genome [10], DNA vaccines failed to emerge in human medicine, although a limited number of vaccines reached the market in veterinary medicine [11] (West-Nile Innovator^®^ DNA, Oncept^®^ Canine Melanoma Vaccine and Elanco’s Clynav^®^ vaccine to control salmon pancreas disease). On the contrary, mRNA vaccines which were not initially actively developed due to important concerns regarding their low stability, gained considerable interest due to the coronavirus disease 2019 (COVID-19) crisis, and are currently close to the market in human medicine. In 2020, many interesting and comprehensive reviews [12,13,14] and original papers [15,16,17,18,19] summarizing the current knowledge on RNA vaccines and presenting some major breakthroughs in their development were published. RNA vaccines have never been so close to the market, and we can reasonably expect to see at least a first one against severe acute respiratory syndrome coronavirus 2 (SARS-CoV-2) infection available by the end of the year, and the beginning of 2021. Indeed, two RNA vaccines against SARS-CoV-2 are currently terminating phase 3 clinical trial with some preliminary reports already published [16,17,19] and many other ones are following. With these new vaccines available soon, there is no doubt we will soon see more RNA vaccines, designed to prevent or even treat various medical conditions, including cancer, on the market. Specifically regarding flu vaccines, one of the biotech company involved in the race for the development of an effective RNA vaccine to prevent COVID-19 had already announced their intention to enter the seasonal flu market given the high medical need for more effective flu vaccines. The interest of RNA vaccines for flu vaccination has also been comprehensively reviewed [20]. Vaccination against influenza viruses faces multiple important challenges that need to be resolved to bring universal and more effective vaccines onto the market [20]. Amongst the commonly described challenges are: (1) the lack of protection of current vaccine formulations against antigenic drift and shift—the two main mechanisms of evolution in Orthomyxoviruses; (2) the short-lived immune response after vaccination; (3) the sometimes weak immune antibody response resulting from pre-existing immunity; (4) potential adverse effects of live attenuated vaccines when they are used; (5) the interference of maternally-derived antibodies (MDA) with the induction of a protective immune response in infants; (6) the common use of adjuvants, especially for inactivated vaccines, not always very well-accepted by the population. Based on the current research, RNA vaccines developed against flu could broadly induce protective immune responses and could overcome some of the issues mentioned above [20,21]. Antigenic drift—RNA segment reassortment resulting from coinfection events—is a major concern for health authorities because it can cause the rapid emergence of potentially pandemic influenza viruses [1,22]. This is why the circulation of influenza viruses between their wild bird reservoirs and some mammalian hosts such as pigs which can act as “mixing vessels” [23] is closely monitored [22]. In the case of emergence, there is an absolute need for the fast development of effective vaccines widely available for the exposed populations. Two of the main advantages of RNA vaccines are that they can be easily updated once the genome sequence of the emerging influenza virus strain has been accurately identified, and they do not require toxic materials or cell cultures that could be contaminated with viruses. Besides these two major advantages, they do not require the systematic use of adjuvants, they do not exhibit any risk of reversion to virulence unlike many attenuated vaccines, and they commonly elicit well-balanced—humoral and cellular—immune responses. Regarding the contemporary drawbacks, a few can be identified. The main one is still the relatively low stability of the RNA—even though progress has been made—and the need for freezer conditions for the distribution and the administration, RNA being more likely to break apart above freezing temperatures [24]. Another limitation is the potential negative impact of type 1 and 3 interferons induced in response to vaccine RNA molecules on antigen expression [24]. Then, even if the risk of genomic integration is widely considered as null and is not a biosafety concern, eukaryotic cells have been shown to be able to provide, to some extent, reverse transcription activity [25,26,27,28]. Further research on that eukaryotic reverse transcription activity, in the context of RNA vaccination, might be of interest for the scientific community [14].

Until now, in vitro transcribed (IVT) messenger RNA (mRNA) influenza virus vaccines were among the most studied RNA vaccines developed against infectious diseases in humans and animals. Two main types of mRNA vaccines have been developed against influenza virus infection: the non-replicating mRNA vaccines and the self-amplifying mRNA vaccines [20], with possibly different types of RNA (prokaryotic, eukaryotic and transfer RNA amongst others) [29]. Both approaches are in the pipelines of the three current major players in the field of RNA vaccines: Moderna Therapeutics (Cambridge, MA, USA) [30], EpiVax (Providence, RI, USA) and CureVac AG (Tübingen, Germany). Moderna, is working on a non-replicating mRNA vaccine with modified nucleosides incorporated associated to lipid nanoparticles (LNP) while CureVac AG, chose a strategy based on sequence-optimized unmodified mRNA–LNP. The EpiVax vaccine which targets highly pathogenic H7N9 subtype influenza virus is currently in phase 1 clinical trial. The impressive acceleration of RNA vaccine research caused by COVID-19 will probably continue to push forward the development of influenza virus RNA vaccines in the coming years. Besides RNA vaccines against flu and COVID-19, many others against rabies [31,32], Zika [33,34], Chikungunya [35], and other pathogens [20] are also in the pipeline at various stages of development.

Data about RNA vaccines are now accumulating very quickly and the first RNA vaccines have been released in the UK on December 2020 (and several countries are following). Are they going to be as effective and as convenient in their use than their competitors based on different approaches (see for instance [36]) sometimes also very innovative and attractive? Will they bring enough advantages compared to other vaccines to be considered as real game changers? We currently—December 2020—still do not know, but we will for sure very soon. It is just a question of time.
